# Direct Oral Anticoagulants Form Thrombus Different From Warfarin in a Microchip Flow Chamber System

**DOI:** 10.1038/s41598-017-07939-6

**Published:** 2017-08-07

**Authors:** Masanobu Ishii, Koichi Kaikita, Miwa Ito, Daisuke Sueta, Yuichiro Arima, Seiji Takashio, Yasuhiro Izumiya, Eiichiro Yamamoto, Megumi Yamamuro, Sunao Kojima, Seiji Hokimoto, Hiroshige Yamabe, Hisao Ogawa, Kenichi Tsujita

**Affiliations:** 1 Department of Cardiovascular Medicine, Kumamoto University Graduate School of Medical Sciences, Kumamoto, Japan; 2 Department of Cardiovascular Medicine, National Cerebral and Cardiovascular Center, Osaka, Japan

## Abstract

Direct oral anticoagulants (DOACs) have low risk of intracranial hemorrhage compared to warfarin. We sought to clarify the different mechanisms responsible for suppression of bleeding events using the Total Thrombus-formation Analysis System (T-TAS), a flow-microchip chamber with thrombogenic surfaces. Blood samples were obtained at Off- and On-anticoagulant (trough) from 120 consecutive patients with atrial fibrillation (warfarin; n = 29, dabigatran; n = 19, rivaroxaban; n = 47, apixaban; n = 25), which were used for T-TAS to compute the area under the curve (AUC) (AR_10_-AUC_30_) in the AR chip, and to measure plasma concentrations of DOACs at On-anticoagulant. In addition, the two-dimensional area covered by thrombi (%) in the capillary was analyzed every 3 minutes after sample applications. The AR_10_-AUC_30_ correlated weakly and negatively with plasma concentrations of DOACs, and the levels at On-anticoagulant were lower in all groups than at Off-anticoagulant. AR_10_-AUC_30_ levels at Off- and On-anticoagulant were identical among the groups. The thrombi areas in early phase were significantly larger in rivaroxaban and apixaban than warfarin and dabigatran groups. The findings suggested that visual analysis of the AR-chip can identify the differential inhibitory patterns of warfarin and DOACs on thrombus formation under flow condition.

## Introduction

Direct oral anticoagulants (DOACs) are used to reduce the risk of stroke in patients with atrial fibrillation (AF), similar to warfarin^1–4^. Bleeding events are sometimes serious complications in AF patients on anticoagulation therapy. However, randomized control studies have shown the efficacy and safety of the DOACs in AF patients. For example, Chatterjee *et al*.^5^ reported that DOACs are associated with an overall low risk of intracranial hemorrhage (ICH). Another group reported that patients treated with rivaroxaban who develop ICH had relatively small hematoma, showed no signs of expansion of hematoma, and had favorable functional and vital outcomes compared to warfarin-associated ICH^6^.

The prothrombin time-international normalized ratio (PT-INR) is widely used to assess the anticoagulant effects of warfarin. Although it is possible to measure blood concentrations of DOACs^7, 8^, there is currently no simple tool available to monitor the effects of DOACs^9^. Recently, the total thrombus-formation analysis system (T-TAS), a microchip-based flow chamber system designed to evaluate whole blood thrombogenicity, was developed as an easy-to-use system for quantitative analysis of thrombus formation. The T-TAS could be useful for monitoring the anticoagulant effects of DOACs and predicting periprocedural bleeding events^10–13^.

The aim of the present study was to determine differences in the anticoagulation patterns of warfarin and DOACs using the T-TAS in patients with AF who had undergone radiofrequency catheter ablation (RFCA).

## Results

### Patient Characteristics

**Figure 1:**
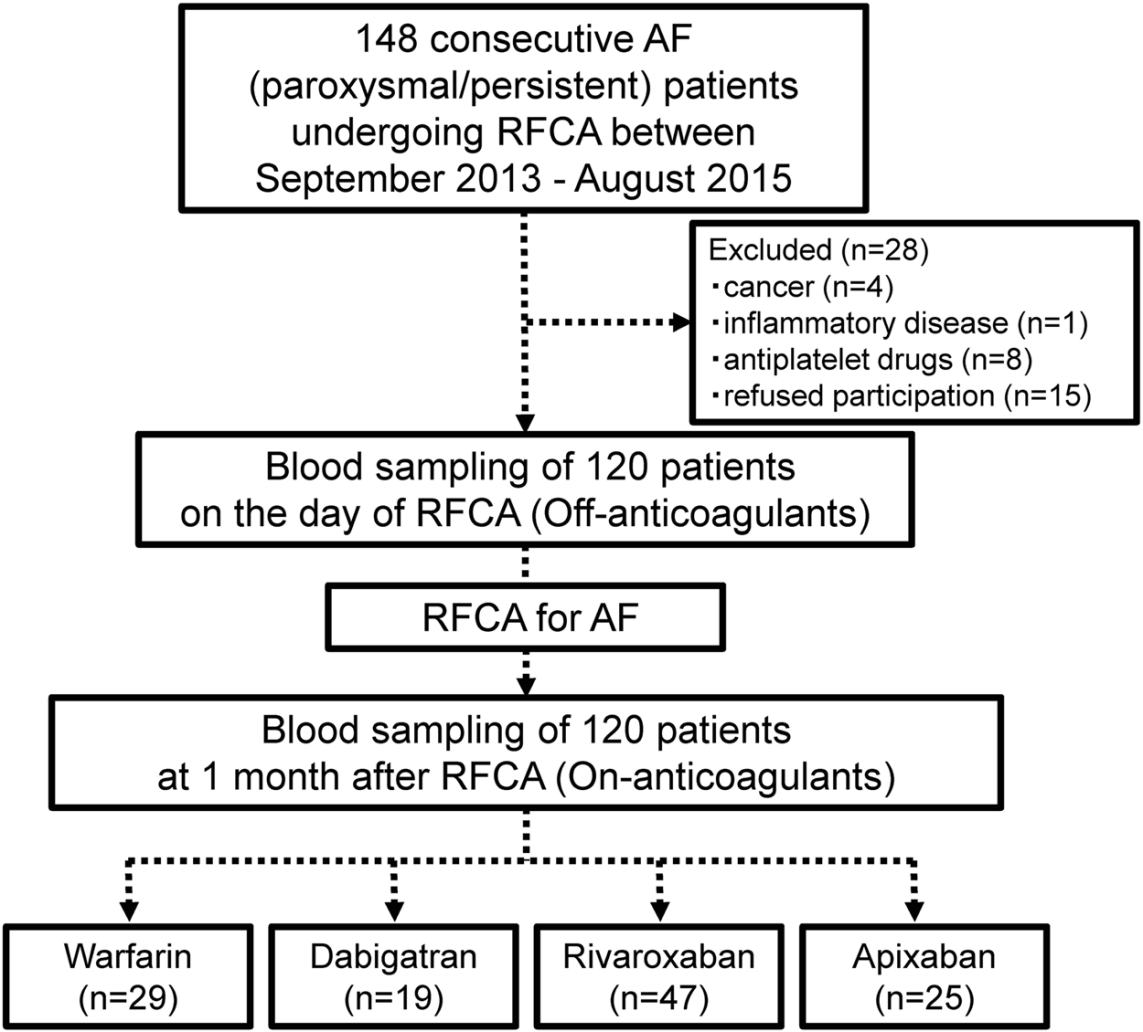
Study flow chart. Chart showing the enrollment criteria and division of patients into different anticoagulant treatment groups. AF = atrial fibrillation, RFCA = radiofrequency catheter ablation.

Figure 1 shows the study flow chart, and Table 1 shows the baseline characteristics of patients treated with warfarin (n = 29), dabigatran (n = 19), rivaroxaban (n = 47), and apixaban (n = 25). There were no significant differences among the groups with regard to age, gender, comorbidity, bleeding risk score, and medications except for the use of antiarrhythmic drugs.

**Table 1:** Comparison of baseline characteristics of patients treated with warfarin and the three types of DOACs.

	Warfarin (n = 29)	Dabigatran (n = 19)	Rivaroxaban (n = 47)	Apixaban (n = 25)	P value
Age (years)	64.6 ± 8.8	60.7 ± 8.2	61.3 ± 9.4	63.1 ± 10.5	0.399
Male (%)	16 (55.2)	14 (73.7)	31 (66.0)	14 (56.0)	0.499
Body weight (kg)	64.9 ± 13.8	64.9 ± 10.5	63.1 ± 10.0	62.5 ± 13.0	0.827
Body mass index (kg/m^2^)	24.4 ± 3.9	23.2 ± 3.3	23.2 ± 2.8	23.5 ± 3.6	0.511
Paroxysmal atrial fibrillation (%)	21 (72.4)	12 (63.2)	34 (72.3)	19 (76.0)	0.819
Duration of atrial fibrillation (months)	24.0 [10.5–75.0]	27.0 [8.8–87.0]	18.5 [6.0–67.3]	11.5 [6.8–48.0]	0.409
Current Smoking (%)	12 (41.4)	6 (31.6)	13 (27.7)	4 (16.0)	0.231
Heart failure (%)	2 (6.9)	1 (5.3)	1 (2.1)	1 (4.0)	0.780
Hypertension (%)	18 (62.1)	12 (63.2)	22 (46.8)	11 (44.0)	0.353
Age ≥ 75 years (%)	3 (10.3)	1 (5.3)	3 (6.4)	5 (20.0)	0.268
Diabetes (%)	2 (6.9)	3 (15.8)	7 (14.9)	3 (12.0)	0.736
Stroke (%)	3 (10.3)	1 (5.3)	4 (8.5)	2 (8.0)	0.942
CHA_2_DS_2_-VASc score	2 [1–3]	2 [1–3]	2 [1–3]	1 [0–3]	0.549
HASBLED score	1 [0–2]	0 [0–1]	1 [0–1]	1 [0–1]	0.171
LAD (mm)	36.3 ± 5.9	37.6 ± 6.3	37.4 ± 5.8	36.5 ± 7.1	0.814
EF (%)	63.0 ± 4.9	62.4 ± 4.7	62.4 ± 6.1	64.9 ± 5.5	0.292
Total bilirubin (mg/dL)*	0.9 ± 0.4	0.8 ± 0.4	0.9 ± 0.3	1.0 ± 0.5	0.277
AST (U/L)*	22 ± 5	23 ± 7	25 ± 7	22 ± 5	0.229
ALT (U/L)*	19 ± 9	20 ± 13	24 ± 13	19 ± 9	0.177
eGFR (mL/min/1.73 m^2^)*	69.3 ± 11.5	71.3 ± 12.0	73.0 ± 12.2	72.8 ± 12.0	0.576
Hb (g/dL)*	14.0 ± 1.5	13.8 ± 1.6	14.1 ± 1.3	14.0 ± 1.4	0.874
Platelet count (10^3^/μL)*	205.5 ± 45.2	214.9 ± 37.8	212.9 ± 38.9	196.8 ± 46.4	0.393
BNP (pg/mL)*	35.6 [16.4–65.0]	33.0 [16.6–95.1]	39.3 [18.2–84.6]	44.1 [19.1–65.1]	0.768
CCB (%)	13 (44.8)	8 (42.1)	18 (39.1)	6 (24.0)	0.421
β-blockers (%)	11 (37.9)	5 (26.3)	18 (38.3)	10 (40.0)	0.784
ARB/ACE-I (%)	10 (34.5)	8 (42.1)	19 (41.3)	7 (28.0)	0.676
Statins (%)	7 (24.1)	3 (15.8)	14 (29.8)	4 (16.0)	0.482
Antiarrhythmic drug (%)	15 (51.7)	18 (94.7)	31 (66.0)	16 (64.0)	0.021
PPI (%)	7 (24.1)	8 (42.1)	9 (19.1)	6 (24.0)	0.278
Dose of DOACs					0.278
Standard dose	—	17 (81.0)	45 (86.5)	24 (96.0)	
Low dose	—	4 (19.0)	7 (13.5)	1 (4.0)	

DOACs = Direct oral anticoagulants, CHA_2_DS_2_-VASc = congestive heart failure, hypertension, age ≥75 years, diabetes mellitus, and prior stroke, transient ischemic attack, or thromboembolism, vascular disease, age 65–74 years, sex category, HASBLED = hypertension, abnormal renal/liver function, stroke, bleeding history or predisposition, labile international normalized ratio, elderly, drugs/alcohol concomitantly, LAD = Left atrial diameter, EF = Left ventricular ejection fraction, AST = aspartate aminotransferase, ALT = alanine aminotransferase, Hb = hemoglobin, eGFR = estimated glomerular filtration rate, BNP = B-type natriuretic peptide, CCB = Calcium channel blocker; ACE-I = angiotensin-converting enzyme inhibitor, ARB = Angiotensin II receptor blocker, PPI = proton pump inhibitor.

^*^Data of these parameters were obtained at admission. Data are expressed as mean ± standard deviation, median [25% to 75%] or n (%). P values are for differences among the four groups by chi-squared test, one-way ANOVA or Kruskal-Wallis test.

### Light Microscope and Fluorescence Images of Thrombus Formation

**Figure 2:**
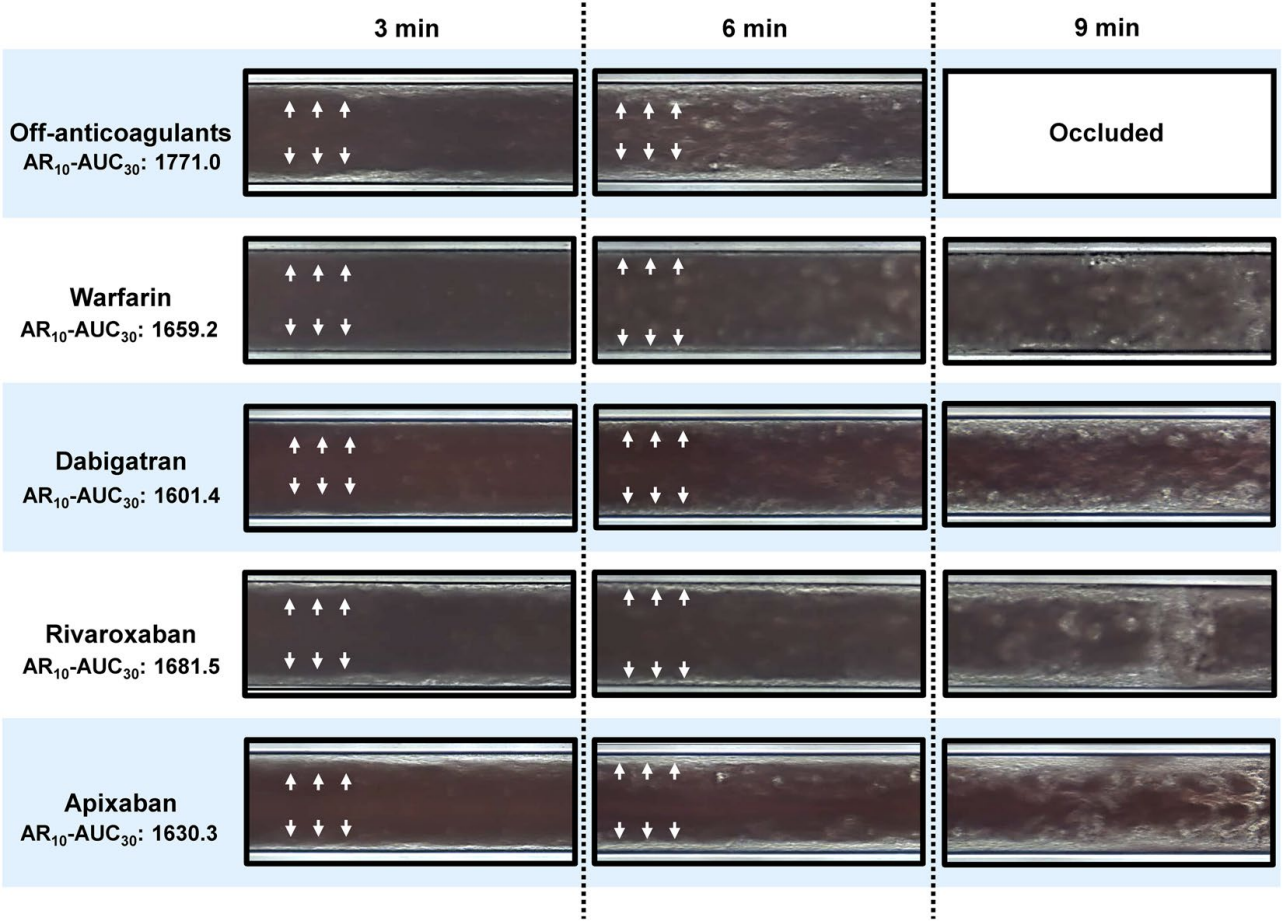
Light microscope images of the AR-chip. Representative microscopic images of thrombus formation under flow condition in Off-anticoagulants, warfarin, and DOAC groups.

Figure 2 shows representative light microscope images of thrombus formation under the flow condition in the AR-chip. Total thrombus formation (indicated in white) was suppressed in both warfarin- and DOACs (dabigatran, rivaroxaban, and apixaban)-treated groups compared to the Off-anticoagulants. Interestingly, thrombi formed at the wall side were thicker in patients treated with rivaroxaban and apixaban (indicated by white arrows), compared with warfarin. On the other hand, thrombi in dabigatran were similar to those in warfarin.

### Effects of Anticoagulants on PT-INR, APTT, and AR_10_-AUC_30_

To evaluate the utility of AR_10_-AUC_30_ measured by T-TAS, we compared the parameters at the Off- and On-anticoagulants. At the Off-anticoagulants, there were no significant differences in PT-INR and AR_10_-AUC_30_ levels between the four groups (Table 2). However, the APTT at Off-anticoagulants in the dabigatran group (34.5 sec) was significantly longer than in the rivaroxaban group (29.9 sec, P = 0.001). On the other hand, at the On-anticoagulants, the PT-INR was significantly longer in the warfarin group than the other groups (P < 0.001, each). APTT was significantly longer in the dabigatran group (42.7 sec) than the rivaroxaban group (33.4 sec, P = 0.001). However, there were no significant differences in AR_10_-AUC_30_ levels among these groups.

**Table 2:** PT-INR, APTT, and T-TAS parameters according to the type of anticoagulant.

	Warfarin (n = 29)	Dabigatran (n = 19)	Rivaroxaban (n = 47)	Apixaban (n = 25)	P value
PT-INR					
Off-anticoagulants	1.06 [1.03–1.12]	1.03 [1.00–1.09]	1.06 [1.01–1.10]	1.05 [1.01–1.09]	0.532
On-anticoagulants	1.91 [1.60–2.20]	1.10 [1.05–1.14]	1.11 [1.05–1.18]	1.16 [1.10–1.23]	<0.001
APTT (sec)					
Off-anticoagulants	31.1 [29.7–34.0]	34.5 [32.1–38.3]	29.9 [28.5–32.9]	31.9 [29.9–34.3]	0.002
On-anticoagulants	38.6 [36.0–44.4]	42.7 [37.9–47.2]	33.4 [31.4–36.5]	33.8 [31.8–36.8]	<0.001
AR_10_-AUC_30_					
Off-anticoagulants	1749 [1646–1824]	1786 [1691–1861]	1812 [1686–1861]	1781 [1673–1861]	0.460
On-anticoagulants	1623 [1385–1706]	1601 [1456–1708]	1657 [1590–1742]	1630 [1469–1771]	0.263
Thrombi area (%)					
3 minutes					
Off-anticoagulants	9.6 [8.2–12.4]	9.2 [7.2–12.0]	10.4 [8.5–12.9]	10.1 [9.0–11.5]	0.351
On-anticoagulants	6.8 [3.3–8.4]	5.6 [4.3–8.4]	8.1 [6.7–11.0]	9.4 [6.2–10.6]	<0.001
6 minutes					
Off-anticoagulants	14.5 [11.2–19.4]	15.5 [11.2–19.2]	17.5 [13.6–20.4]	15.3 [13.5–18.1]	0.574
On-anticoagulants	9.9 [6.5–13.4]	10.0 [7.3–13.4]	13.6 [10.9–17.0]	13.9 [11.3–18.0]	<0.001
9 minutes					
Off-anticoagulants	18.7 [15.1–22.7]	23.4 [19.2–27.9]	21.5 [14.3–27.4]	22.0 [18.2–23.9]	0.373
On-anticoagulants	17.2 [9.4–22.2]	17.8 [13.3–21.4]	18.7 [15.5–24.6]	20.8 [15.3–24.1]	0.077

Data are median [25% to 75%].

P values are for differences among the four groups by Kruskal-Wallis test.

**Figure 3:**
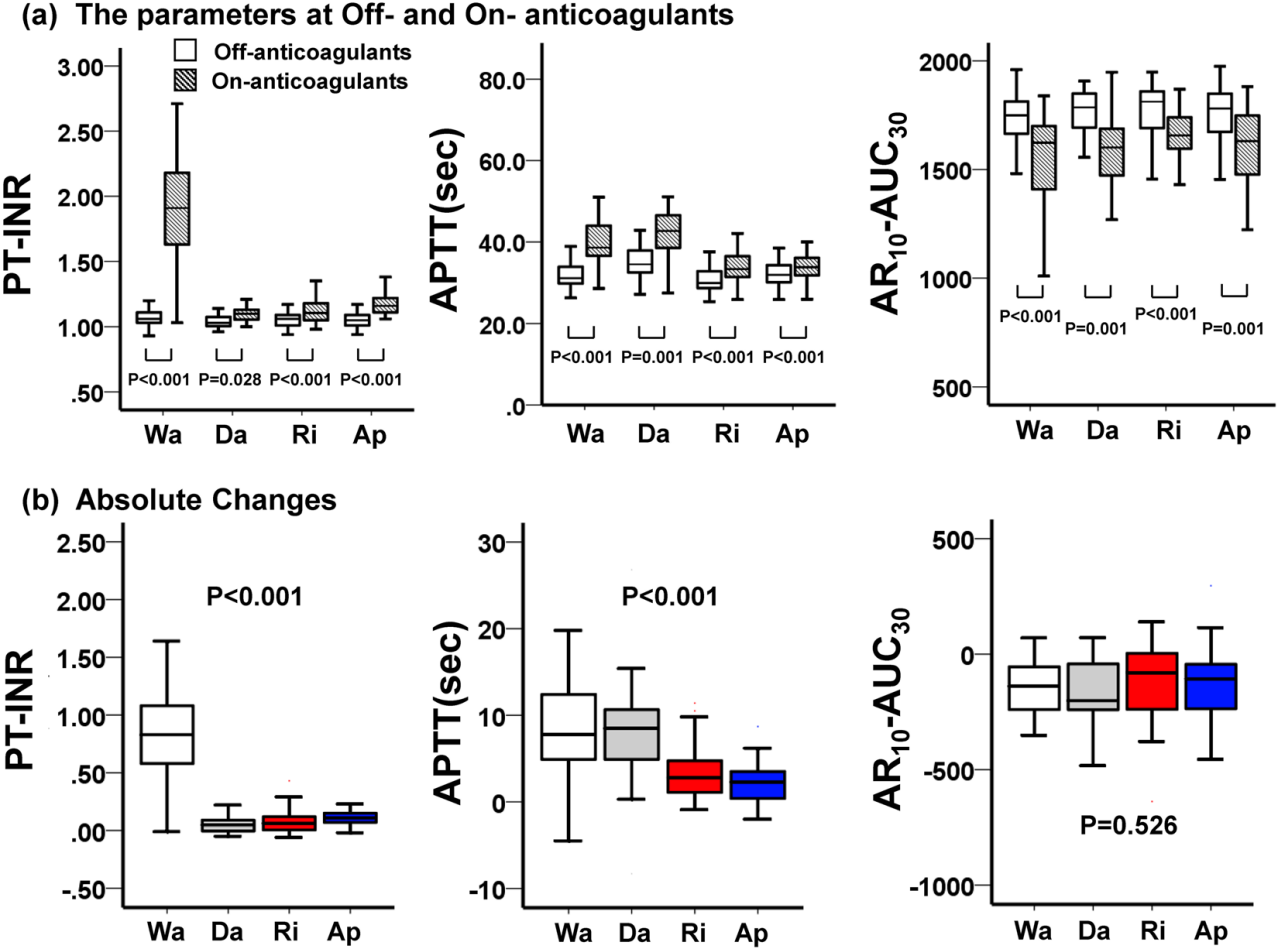
Quantitative analysis of various parameters at Off- and On-anticoagulants. In these box-and-whisker plots, lines within the boxes represent median values; the upper and lower lines of the boxes represent the 25th and 75th percentiles, respectively; and the upper and lower bars outside the boxes represent maximum and minimum values, respectively. Wa = warfarin, Da = dabigatran, Ri = rivaroxaban, Ap = apixaban

AR_10_-AUC_30_ levels were significantly lower at On-anticoagulant than at Off-anticoagulant, whereas PT-INR and APTT were significantly longer at On-anticoagulant than Off-anticoagulant (Fig. 3a). In addition, we analyzed the absolute changes in PT-INR, APTT, and AR_10_-AUC_30_ (Fig. 3b). The changes in PT-INR and APTT were different among the four groups, however; changes in AR_10_-AUC_30_ were not significantly different among the groups, indicating that AR_10_-AUC_30_, but not PT-INR and APTT, can reflect the anticoagulative effects of each drug uniformly.

### Relationship Between AR_10_-AUC_30_ and Plasma Concentrations of DOACs

**Figure 4:**
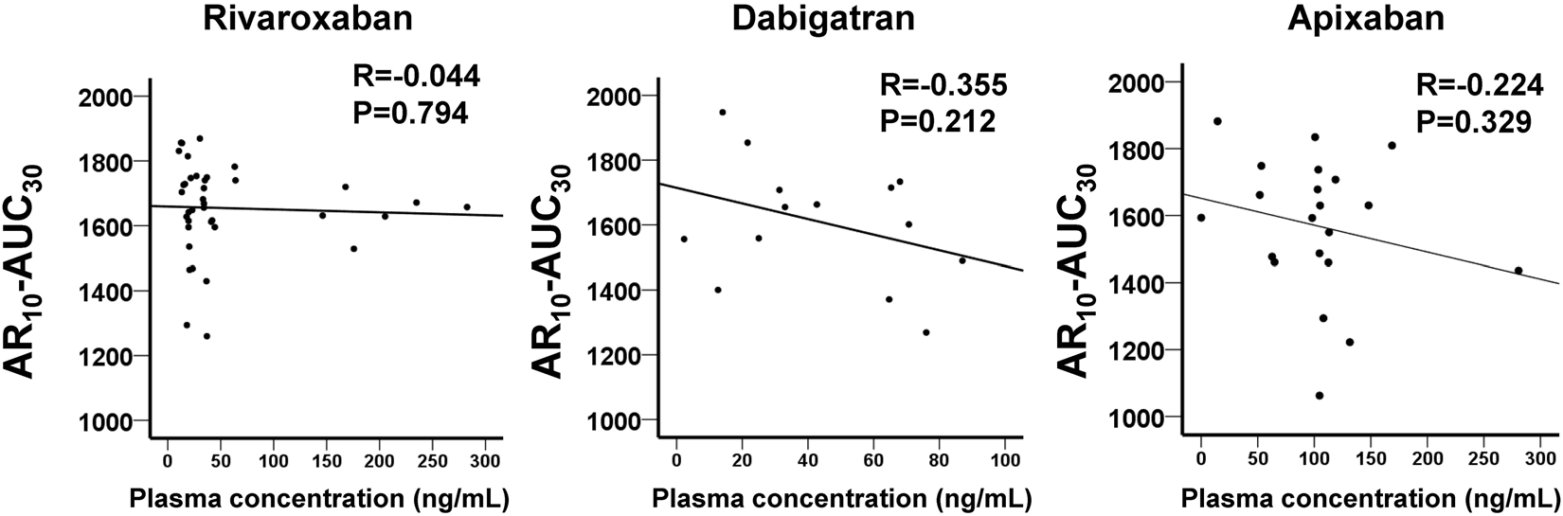
Scatter plots of AR_10_-AUC_30_ levels and plasma concentrations of DOACs. Relationship between AR_10_-AUC_30_ levels measured by T-TAS and plasma concentrations of rivaroxaban, dabigatran and apixaban measured by the respective coagulation assays at On-anticoagulants.

To evaluate the utility of AR_10_-AUC_30_ measured by T-TAS, we compared the levels of AR_10_-AUC_30_ and plasma concentrations of DOACs at On-anticoagulants (trough). Figure 4 shows scatter plots for the relation between AR_10_-AUC_30_ levels and plasma concentrations in the three DOAC groups. There was almost no relation between AR_10_-AUC_30_ and plasma concentrations of rivaroxaban (n = 38, R = −0.044). On the other hand, there was a weak negative relation between AR_10_-AUC_30_ and dabigatran (n = 14, R = −0.355) and apixaban (n = 21, R = −0.224).

### Effects of Anticoagulants on Area of Thrombi

**Figure 5:**
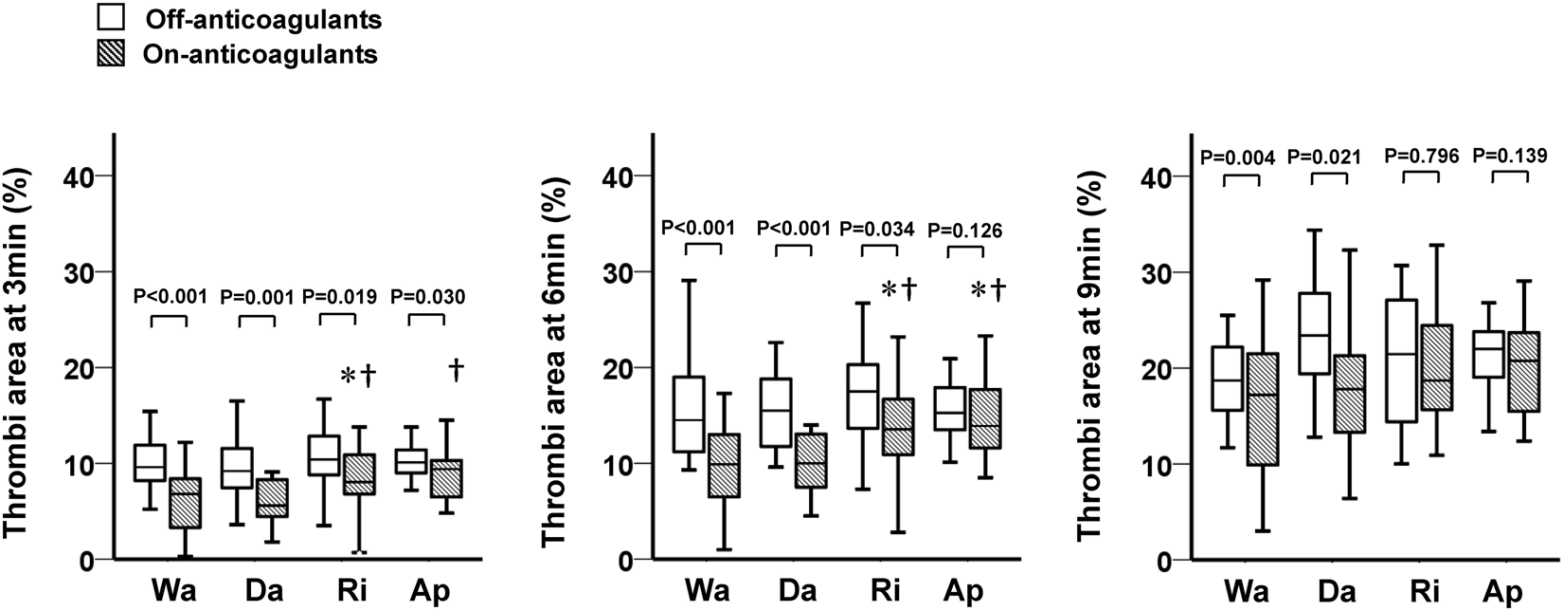
Thrombi area computed from analysis of the AR chip induced by various anticoagulants. In these box-and-whisker plots, lines within the boxes represent median values; the upper and lower lines of the boxes represent the 25th and 75th percentiles, respectively; and the upper and lower bars outside the boxes represent maximum and minimum values, respectively. *Adjusted P < 0.05 vs. warfarin at On-anticoagulants; significance probability was adjusted for Bonferroni method. †Adjusted P < 0.05 vs. dabigatran at On-anticoagulants; significance probability was adjusted for Bonferroni method. Wa = warfarin, Da = dabigatran, Ri = rivaroxaban, Ap = apixaban.

To investigate the different thrombus formation under flow condition in the four groups, we examined thrombus formation inside the AR-chip by the built-in light microscope. AR_10_-AUC_30_ levels at On-anticoagulants were identical among the four medications drug (Table 2). However, the thrombi area was significantly different in the early phase of thrombus formation (3 and 6 min) among the four groups. The thrombi areas at 3 and 6 min were significantly larger in the rivaroxaban group than the warfarin and dabigatran groups (Fig. 5). Similarly, the thrombi area at 3 min in the apixaban group was significantly larger than in the dabigatran group, and tended to be larger than in the warfarin group (p = 0.052). The thrombi area at 6 min was significantly larger in the apixaban group compared to the warfarin and dabigatran groups, similar to the rivaroxaban group. At 9 minutes, there were no significant differences in the thrombi area at On-anticoagulants among the groups.

The thrombi area at 3 min was significantly smaller at On-anticoagulants than Off-anticoagulants in all groups (Fig. 5). However, the thrombi area at 6 min was not significantly different between On- and Off-anticoagulants in the apixaban group. The same was also true for those at 9 min between the rivaroxaban and apixaban groups.

## Discussion

The major findings of this study were as follows: (1) AR_10_-AUC_30_ could be a useful marker for monitoring the anticoagulant effects of warfarin and DOACs, although it had weak negative correlation with plasma concentrations of DOACs; and (2) the area of thrombi during the early phase of thrombus formation was significantly larger in patients treated with rivaroxaban and apixaban compared to those on warfarin and dabigatran, indicating that these differential patterns of thrombus formation might explain the mechanism of volume reduction of ICH. To the best of our knowledge, this is the first report that determined the differential mechanism of antithrombotic effects in patients treated with warfarin and DOACs by using T-TAS, a novel quantitative assessment of whole-blood thrombogenicity.

Monitoring of the anticoagulant effects of warfarin requires the measurement of PT-INR to determine the optimal dose, but there is no available tool for the monitoring of DOACs^9^. Several previous studies reported that dabigatran prolonged APTT in a concentration-dependent manner^14, 15^, rivaroxaban prolonged PT in a concentration-dependent manner^16, 17^, and apixaban prolonged PT and APTT, but their correlation was modest or weak^18, 19^. In the present study, dabigatran, rivaroxaban, and apixaban significantly prolonged PT-INR and APTT, though their effects were not identical. Therefore, it could be difficult to set the optimal therapeutic range of using the PT-INR and APTT for the anticoagulant therapy with DOACs. However, AR_10_-AUC_30_ levels measured by T-TAS were uniformly lower under treatment by both DOACs and warfarin, indicating that the AR_10_-AUC_30_ level can reflect the anticoagulant effects on any anticoagulant therapy with an easy-to-use procedure.

Measurement of plasma concentrations is often used for monitoring DOACs, and several coagulation assays have been developed for monitoring of treatment with DOACs^7, 8^. To investigate the relationship between AR_10_-AUC_30_ levels and plasma concentrations, we measured the latter employing coagulation assays. Analysis of the relationship between AR_10_-AUC_30_ levels and plasma concentrations showed almost no relation in patients treated with rivaroxaban, and weak relation in dabigatran and apixaban. The reason for this finding might be that the overall coagulability depends on not only the plasma concentration of DOAC, but also other coagulation factors, old age, and presence of pathological conditions (e.g., congestive heart failure, renal failure, pancytopenia, cancer). In a previous study from our laboratories, although the number of patients was relatively small, statistical analysis identified the AR_10_-AUC_30_ level measured by T-TAS as a potentially useful predictor of periprocedural bleeding events in AF patients undergoing RFCA^12^. Especially, in the evaluation of bleeding risk of patients treated with oral anticoagulants, assessment of total coagulability is probably more important than measurement of plasma concentrations of DOACs alone. Further prospective studies of large population are needed to establish the utility of AR_10_-AUC_30_ level for monitoring the anticoagulant effects and the risk of bleeding events in patients treated with warfarin or DOACs.

One previous meta-analysis showed that the use of DOACs is associated with reduced risk of ICH compared to warfarin^5^. The authors hypothesized that the finding indicated different mechanisms of action for DOACs and warfarin, and that the unique physiology of the brain, such as tissue factor-rich basal membrane, provides protection against spontaneous intracerebral hematoma formation^20^. While these mechanisms were supported by the results of several animal studies^21, 22^, there are no clinical studies that analyzed those mechanisms. In the present study, we used an AR-chip with a surface coated with collagen plus tissue factor, for analysis of thrombus formation under flow conditions, and examined the formation of platelet thrombi, which was calculated as the relative area of thrombi. Interestingly, the thrombi area during the early phase (i.e., at 3 and 6 min) was quite different among the four treatment groups, although there were no significant differences in the final AR_10_-AUC_30_ level measured by the T-TAS. The thrombi area at 3 or 6 min was significantly larger in rivaroxaban and/or apixaban (direct inhibitors of coagulation factor Xa) than dabigatran (direct inhibitor of thrombin) and warfarin (reduction of thrombin, factor VII, IX, and X). These findings suggest that thrombin inhibition or decrease seems to markedly reduce the thrombi area during the early phase of the measurement by the AR-chip, whereas factor Xa inhibition does not diminish it significantly, and could reflect the protective mechanism of factor Xa inhibitors against ICH clinically. Why was the thrombi area smaller in dabigatran than in factor Xa inhibitors (rivaroxaban and apixaban)? Against this observation, dabigatran and rivaroxaban have comparable bleeding risk in patients with AF even in real-world practice^23, 24^. In addition, previous experimental studies showed that direct thrombin inhibitors, but not factor Xa inhibitors, enhance thrombin generation paradoxically via intrinsic pathway or inhibition of the protein C system^25, 26^. These discrepancies might be explained by potential mechanism, such as the influence of CaCl_2_ and corn trypsin inhibitor in the collection tubes, and difference in the rivaroxaban dose used clinically in Japan and western countries. However, the clinical relevance of this observation is not clear and it should be evaluated in future clinical studies.

The present study has several limitations. First, the results could be overestimated based on the small sample size in this single-center observational study. Second, the long-term outcome (thrombotic and bleeding events) was not evaluated in AF patients treated with anticoagulants. A large population study is needed to evaluate the relationship between T-TAS parameters (AR_10_-AUC_30_ and thrombi area) and clinical outcome. Thirds, the parameters of RFCA cannot be strictly regarded as ‘off-anticoagulation’ since bridging anticoagulation with unfractionated heparin is used before RFCA in some patients. Finally, the pharmacokinetics and pharmacodynamics need to be analyzed when considering the factors that can influence the effects of anticoagulation. These factors can include for example the activities of drug-metabolizing enzymes and any genetic polymorphism. Such confounding factors, which were not measured in the present study, might limit the significance of the present study due to the retrospective nature of the study design.

In conclusion, the present study demonstrated that AR_10_-AUC_30_ measured by T-TAS is a potentially useful tool for monitoring the anticoagulant effects of warfarin and DOACs. The results also indicated that visual analysis of the obtained microscopic images of the AR-chip could help us understand one of the different mechanisms of hemostatic thrombus formation in patients treated with warfarin and DOACs. However, further investigations are needed to confirm whether the present findings are responsible for the different mechanisms for the suppression of bleeding events between patients treated with warfarin and DOACs.

## Methods

### Study Population

The present study was a subanalysis of a previous study^12^. We enrolled 148 consecutive patients who were ≥20 years of age and had undergone RFCA for AF at Kumamoto University Hospital between September 2013 and August 2015. We excluded 28 patients for the following reasons: cancer (n = 4), inflammatory disease (n = 1), use of antiplatelet drugs (n = 8), and refused participation (n = 15). The remaining 120 patients treated with anticoagulants were the subjects of this study. They were divided into four treatment groups according to type of anticoagulant used; warfarin (n = 29), dabigatran (n = 19), rivaroxaban (n = 47), and apixaban (n = 25) groups (Fig. 1). Based on the current guideline and the package insert of each anticoagulant in Japan, we prescribed a low dose of each DOACs: dabigatran 110 mg twice daily for patients ≥70 years of age or those with creatinine clearance (Ccr) of 30–50 mL/min; rivaroxaban 10 mg once daily with Ccr 15–49 mL/min; apixaban 2.5 mg twice daily when two of the following three criteria were identified: age ≥80 years, body weight ≤60 kg, or creatinin ≥1.5 mg/dL. The dosage of warfarin was adjusted to maintain INR at 2.0–3.0 (age <70 years) or 1.6–2.6 (age ≥70 years).

All procedures were conducted in accordance with the Declaration of Helsinki and its amendments. The study protocol was approved by the Human Ethics Review Committee of Kumamoto University, and written informed consent was obtained from each patient or the family of the subject.

### Collection of Blood Samples

Details of the blood sampling points and the washout period of anticoagulants during RFCA were described previously^12^. Briefly, DOACs (dabigatran, rivaroxaban, and apixaban) were stopped in the morning the day before RFCA, and were bridged with unfractionated heparin until 6 hours before RFCA. Warfarin was interrupted 4 days before RFCA, and bridged with unfractionated heparin, similar to DOACs. All anticoagulants were restarted from the next morning after RFCA, but warfarin was bridged with unfractionated heparin from the next morning after RFCA until the target PT-INR of 1.6 to 2.6. During the bridging anticoagulation with unfractionated heparin, the heparin dose was adjusted so as to maintain the APTT level at about 1.5 to 2.5 times the baseline level.

We analyzed blood samples obtained on the day of RFCA (Off-anticoagulants point), and 1 month after RFCA (trough, On-anticoagulants point).

### Measurement of Plasma Concentration of DOACs

To investigate the relation between AR_10_-AUC_30_ and plasma concentration of DOACs, we measured plasma concentration of DOACs at On-anticoagulants (at trough) using the commercially available standardized assay kits (Hyphen Biomed, Aniara, West Chester, OH). For rivaroxaban and apixaban, the BIOPHEN DiXaI chromogenic assays were used, as well as the BIOPHEN HEMOCLOT Thrombin Inhibitors clotting assay for dabigatran, according to protocols recommended by the manufacturers.

### Measurement of Thrombogenicity by the T-TAS

We used the T-TAS (Fujimori Kogyo Co., Tokyo, Japan), which is a microchip-based flow chamber system equipped with a flow pressure sensor and a videomicroscope, for the analysis of thrombus formation under flow, by computing the area under the flow/pressure curve (AUC), as described previously^10, 11, 27–29^. Briefly, the atheroma chip (AR-chip) of the T-TAS, which contains a single capillary channel (width 300 μm, depth 80 μm), is coated with type I collagen plus tissue thromboplastin. Whole blood collected into 3.2% sodium citrate-containing tubes was blended with CaCl_2_ and corn trypsin inhibitor immediately before the assay. The 450-μL mixture was applied to the AR-chip at a flow rate of 10 μL/min, corresponding to an initial wall shear rate of 600 s^−1^. Both the collagen and tissue thromboplastin activate the platelets and the coagulation system simultaneously inside the AR-chip. The fibrin-rich platelet thrombus formation processes were assessed by continuous monitoring of the flow/pressure change resulting from capillary occlusion. The AUC was computed to evaluate platelet thrombogenicity in the AR-chip. AR_10_-AUC_30_ is defined as the AUC for the first 30 minutes for the AR-chip tested at flow rate of 10 μL/min. The absolute changes in the quantity of AR_10_-AUC_30_, PT-INR, and activated partial thromboplastin time (APTT) was calculated by the following formula:

$${\rm{Absolute}}\,{\rm{change}}=({\rm{level}}\,{\rm{at}}\,\text{On}-\text{anticoagulants})-({\rm{level}}\,{\rm{at}}\,\text{Off}-\text{anticoagulants})$$

In addition to the flow/pressure analysis, thrombus formation in the capillary was visually observed by the built-in light microscope (Fig. 2), and the two-dimensional area covered by thrombi was computed using an image analysis software (Zia; Fujimori Kogyo Co.), as described previously^28^. The thrombus formation process inside the microchip was recorded at 3, 6, 9 minutes after application of the blood sample.

### Statistical Analysis

Data are expressed as mean ± SD or median. Differences between parameters were compared with the chi-square test, Fisher’s exact test, one-way ANOVA or Kruskal-Wallis test, as appropriate. We also used the Wilcoxon signed-rank test to assess the serial changes in AR10-AUC30, PT-INR, APTT, and the relative area of thrombi (%). A two-tailed *P* value of <0.05 denoted the presence of a statistically significant difference. All statistical analyses were performed by using The Statistical Package for Social Sciences software version 23.0 (IBM Corporation, Armonk, NY).
